# Anionic Phospholipids Induce Conformational Changes in Phosphoenolpyruvate Carboxylase to Increase Sensitivity to Cathepsin Proteases

**DOI:** 10.3389/fpls.2019.00582

**Published:** 2019-05-09

**Authors:** Jacinto Gandullo, José-Antonio Monreal, Rosario Álvarez, Isabel Díaz, Sofía García-Mauriño, Cristina Echevarría

**Affiliations:** ^1^Departamento de Biología Vegetal, Facultad de Biología, Universidad de Sevilla, Seville, Spain; ^2^Centro de Biotecnología y Genómica de Plantas, Universidad Politécnica de Madrid, Campus de Montegancedo, Pozuelo de Alarcón, Madrid, Spain

**Keywords:** phosphoenolpyruvate carboxylase, phospholipids, phosphatidic acid, proteolysis, conformational changes, cathepsin proteases, sorghum

## Abstract

Phosphoenolpyruvate carboxylase (PEPC) is a cytosolic, homotetrameric enzyme that serves a variety of functions in plants, acting as the primary form of CO_2_ fixation in the C_4_ photosynthesis pathway (C_4_-PEPC). In a previous work we have shown that C_4_-PEPC bind anionic phospholipids, resulting in PEPC inactivation. Also, we showed that PEPC can associate with membranes and to be partially proteolyzed. However, the mechanism controlling this remains unknown. Using semi purified-PEPC from sorghum leaf and a panel of PEPC-specific antibodies, we analyzed the conformational changes in PEPC induced by anionic phospholipids to cause the inactivation of the enzyme. Conformational changes observed involved the exposure of the *C*-terminus of PEPC from the native, active enzyme conformation. Investigation of the protease activity associated with PEPC demonstrated that cysteine proteases co-purify with the enzyme, with protease-specific substrates revealing cathepsin B and L as the major protease species present. The anionic phospholipid-induced C-terminal exposed conformation of PEPC appeared highly sensitive to the identified cathepsin protease activity and showed initial proteolysis of the enzyme beginning at the *N*-terminus. Taken together, these data provide the first evidence that anionic phospholipids promote not only the inactivation of the PEPC enzyme, but also its proteolysis.

## Introduction

C_4_-phosphoenolpyruvate carboxylase (PEPC; EC 4.1.1.31) catalyzes the first carboxylation step in C_4_ photosynthesis. Due to the key role that this enzyme plays in the C_4_ pathway, the biochemical and signaling mechanisms controlling PEPC activity in the cytosol of leaf mesophyll cells have received significant research attention (Chollet et al., 1996; Echevarria and Vidal, 2003; Izui et al., 2004). In sorghum, PEPC belongs to a multigene family encoding five closely related plant-type PEPCs (PTPC; SbPEPC1-5) and one distantly related bacterial-type PEPC (BTPC) (Ruiz-Ballesta et al., 2016). Of the PTPCs, one family member, SbPEPC1, is an example of a C_4_ photosynthetic PEPC, playing a functional role in C_4_ and Crassulacean acid metabolism (CAM)-type photosynthesis, while the remaining four homologs, SbPEPC2-5, are C_3_ PEPCs, that perform alternative functions in plants (O’Leary et al., 2011). C_4_-PEPC has evolved from an ancestral non-photosynthetic C_3_-PEPC. Two important aa determine C_3_/C_4_-specific function. In the susbtrate-binding center, Ala774 (*Flaveria* numbering) mediates C_3_ specificity, while Ser774 determines the increased kinetic efficiency of C_4_ PEPC (Paulus et al., 2013). Malate binding in the inhibitory site, is controlled by Arg884 in the C_3_ enzyme while, the increased tolerance of C_4_-PEPC to the malate inhibitor is mediated by Gly884 (Paulus et al., 2013). All PTPCs are subject to a light-dependent phosphorylation process involving an *N*-terminal regulatory serine that interacts with metabolite effectors, such as the feedback inhibitor L-malate, and the allosteric activator Glucose-6-phosphate (Glc-6P; Echevarria and Vidal, 2003). In addition, C_3_-type plant PEPCs have also been shown to be regulated by monoubiquitination (Uhrig et al., 2008; Shane et al., 2013; Ruiz-Ballesta et al., 2014, 2016), though this has not been demonstrated for the photosynthetic C_4_-PEPC to date (unpublished results).

C_4_-PEPC, Rubisco and pyruvate orthophosphate dikinase are very abundant in plants with C_4_-photosynthesis and together account for almost the same amount of protein as observed for Rubisco alone in C_3_ plants (Feller et al., 2008). While numerous studies have described Rubisco proteolysis occurring as a consequence of senescence and nutrient reutilization (Otegui, 2018), little is known about the mechanism by which C_4_-PEPC may be degraded. Scarce data on the protease(s) involved in determining the C_4_-PEPC turnover rate and the amount of protein in the leaf tissue under normal and stress conditions are available. A study by Monreal et al. (2007), showed that a decrease in C_4_-PEPC leaf content occurred in response to LiCl treatment and senescence. However, the integrity and activity of the remaining enzyme were unaffected, suggesting reduced catabolism of C_4_-PEPC in response to these conditions (Monreal et al., 2007). A variety of proteolytic activities have been proposed for the degradation of other PEPC variants in different plant cell types. It has been hypothesized that the proteolysis of C_3_-PEPC in guard cells during their closing phase of action may be dependent on the ubiquitin-proteasome system (Klockenbring et al., 1998). In addition, a C_3_-PEPC in castor oil seeds is degraded *in vitro* via a thiol endopeptidase requiring dithiothreitol and salt to remove its *N*-terminus thereby generating a 98 kDa PEPC fragmented form (Crowley et al., 2005; Uhrig et al., 2008). Finally, a bacterial PEPC from castor oil seeds has been shown to be degraded via a cysteine endopeptidase (Gennidakis et al., 2007).

We previously reported that C_4_-PEPC, from sorghum, is a phosphatidic acid (PA)-binding protein that shows inhibited activity in presence of PA and other anionic phospholipids (Monreal et al., 2010). In comparison, a study examining tomato and Arabidopsis plants, demonstrated that C_3_-PEPC isoenzymes react differently to their C_4_-PEPC counterparts, binding PA in cell cultures with increased affinity under conditions of cellular stress (Testerink et al., 2004). However, the molecular basis for this difference in C_4_- and C_3_-PEPC isoenzymes requires further investigation. Monreal et al. (2010) have shown, via fractionation of sorghum leaf crude extracts, that while the majority of the enzyme is present in the cytosol, a portion of C_4_-PEPC is also associated with the cell membranes where it was found to be partially degraded (Monreal et al., 2010). This finding paves the idea that the interaction of PEPC with the membrane phospholipids could indeed lead to its degradation.

In this study we used antibodies to the final 19 C-terminal amino acids of PEPC (C19-IgG) to investigate the effects of the anionic phospholipids PA, phosphatidylinositol 4-P (PI), and lyso-PA (LPA) on PEPC and their potential to induce a conformational change in the enzyme, exposing the *C*-terminus and inactivating the complex. The *C*-terminal domain of PEPC is normally embedded in a hydrophobic region of the protein subunit (Kai et al., 1999; Alvarez et al., 2003), where the carboxyl group of the *C*-terminal residue G960 moves toward the PEP-binding site to allow formation of an ion pair, with the side chain of R647, forming the active enzyme conformation (Kai et al., 2003). The exposure of the *C*-terminus promoted by anionic phospholipids may disrupt the interaction of the ion pair with the R647 side chain, resulting in the inactivation of the enzyme. In addition, this exposed *C*-terminus conformation is highly sensitive to proteolysis by cysteine proteases, which co-purified with PEPC. Here we examine the precise role of PA and anionic phospholipids in PEPC proteolysis and partial characterization of the papain proteases co-purified with PEPC.

## Materials and Methods

### Materials

Polyclonal antibodies against: (i) synthetic peptides corresponding to sorghum C_4_-PEPC *C*-terminus [(Y) 942EDTLILTMKGIAAGMQNTG960] and dephosphorylated *N*-terminus [4ERHHSIDAQLRALAPGKVSEE24(YG)], C19-IgG and N24-IgG, respectively, were purchased from NEOSYSTEM S.A. (Strasbourg, France); and (ii) native C_4_-PEPC from sorghum leaves (PEPC-IgG) achieved as described in Pacquit et al. (1995).

Phospholipids with varying format and fatty acyl chain composition were all purchased from Avanti Polar Lipids, (Alabaster, AL, United States). Lipids in chloroform stock were dried with gaseous N_2_, rehydrated in 0.1 M Tris–HCl buffer (pH 8) and sonicated prior to adding to the assays. Protease inhibitors used were: Chymostatin (cysteine protease, chymotrypsin and elastase inhibitor); phenylmethane sulfonyl fluoride (PMSF; serine protease inhibitor); Aprotinin (serine protease inhibitor); Bestatin (aminopeptidase inhibitor); Leupeptin (serine and cysteine protease inhibitor); E-64 (selective cysteine protease inhibitor, such as cathepsin B and L); and protease inhibitor cocktail (AEBSF; serine proteases inhibitor; Phenanthroline, metalloproteases inhibitor; Pepstatin A, acid proteases inhibitor, Leupeptine, Bestatin and E-64) from Sigma Aldrich code P9599 (St Louis, MO, United States).

### Plant Material and Growth Conditions

Sorghum plants (*Sorghum bicolor* (L.) Moench, var. PR87G57; Pioneer Hi-Bred Spain) were grown under controlled environmental conditions in a greenhouse, using a 12 h photoperiod (350 μmol m^-2^ s^-1^, photosynthetically active radiation), a temperature of 28/20°C (light/dark) and 60% relative humidity, in hydroponic cultures with nitrate-type nutrient solution (Hewitt, 1966).

### Preparation of Semi-Purified C_4_-PEPC Fraction

All procedures were carried out at 4°C. Dark-adapted (12 h) sorghum leaves (20 g) were homogenized in a Waring blender with 100 mL of extraction buffer containing 0.1 M Tris–HCl pH 7.5, 5 % (v/v) glycerol, 1 mM EDTA, 10 mM MgCl_2_ and 14 mM β-mercaptoethanol, 1 mM phenylmethysulfonyfluoride (PMSF), 10 μg mL^-1^ chymostatin, 10 μg mL^-1^ leupeptin, 10 mM potassium fluoride, and 2% (w/v) polyvinylpyrrolidone (PVP). The homogenate was filtered through two layers of 80 μm nylon net and centrifuged at 45,000 *g* for 10 min. Proteins in the supernatant were precipitated by polyethylene glycol 8000 (PEG; 8.5% – 15%) and then sedimented by centrifugation (45,000 *g*, 10 min). The pellet was dissolved in 7 mL of buffer A containing 50 mM Hepes/ KOH pH 7.1, 5 mM MgCl_2_, 1 mM EDTA, 5 mM dithiothreitol (DTT). Econo-Pac CHT-II chromatography cartridges packed with hydroxyapatite (5 mL) from Bio-Rad (Berkeley, CA, United States) were equilibrated with buffer A and anion-exchange chromatography (5 mL, Bio-Rad catalog number 723-4122) was performed according to the procedure of McNaughton et al. (1989), with the exception that chromatography was performed in a Bio-Rad Econo-System at low pressure. The final specific activity of the semi-purified fraction was determined to be 78.5 ± 5 U ml^-1^. PEPC activity and L-malate sensitivity were determined as described in Echevarria et al. (1994). Soluble protein concentration was measured via a Bradford assay (Bradford et al., 1976), using bovine serum albumin (BSA) as a standard. This final preparation was stored at -20°C, in the presence of 50% glycerol and used as a semipurified-PEPC (sp-PEPC).

### Preparation of Crude Extracts From Sorghum Leaves

Protein extracts were obtained by grinding 0.2 g fresh weight of leaf tissue using sand and 1 ml of extraction buffer containing: 0.1 M Tris–HCl buffer (pH 7.5), 20% (v/v) glycerol, 1 mM EDTA, 10 mM MgCl_2_, 14 mM β-mercaptoethanol, 1 mM PMSF, 10 μg mL^-1^ leupeptin. The homogenate was centrifuged at 12,000 *g* for 2 min. The supernatant was removed and used as a clarified protein extract.

### Assessment of Phospholipid Activity on PEPC Activity

**Figure 1:**
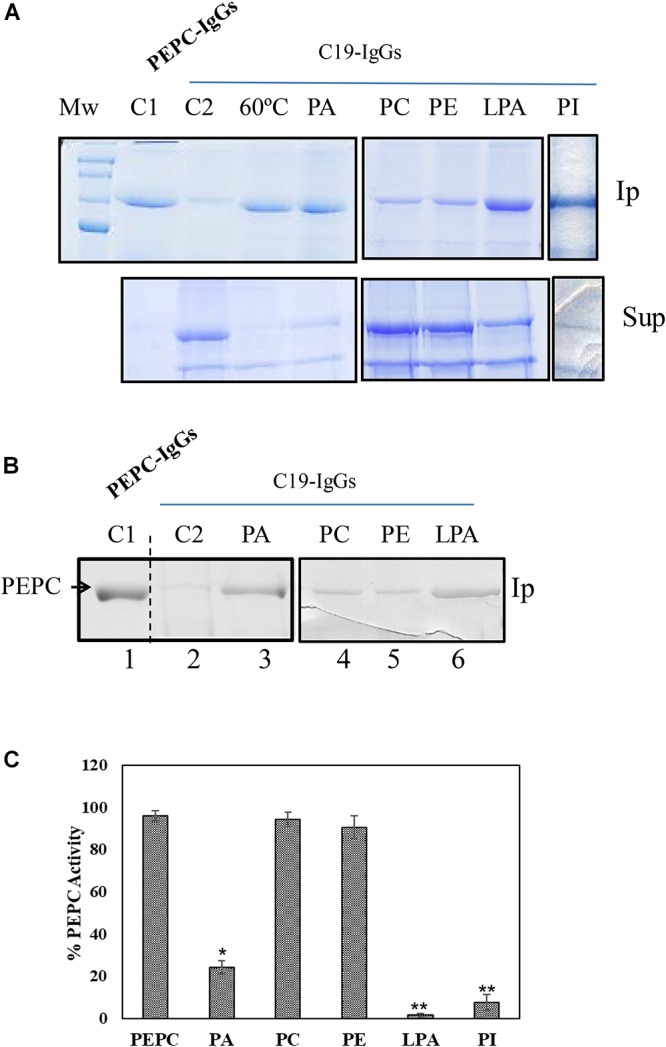
Anionic phospholipids promoted extensive conformational changes in PEPC detected by the exposure of its *C*-terminus. 0.2 U **(A)** or 0.1 U **(B)** of PEPC were incubated in the absence (lanes C1, C2 and 60°C) or presence of 0.25 mM of the phospholipids indicated (lanes PA, PC, PE, LPA, PI). Following 30 min incubation at 30°C, PEPC was immunoprecipitated with PEPC-IgG (lane C1) or C19-IgG (remaining samples). An aliquot of PEPC was heated at 60°C for 2 min to serve as a positive control for C19-IgG (lane 60°C). This treatment completely exposes the *C*-terminus of the protein (Alvarez et al., 2003). **(A)** Immunoprecipitates (Ip) and supernatant (Sup) were analyzed by SDS–PAGE (10%) and stained with Coomassie blue. **(B)** Immunoprecipitates were immunoblotted with PEPC-IgG. The images in **(A)** and **(B)** are representative of at least 3 independent experiments. **(C)** Effect of the different phospholipids on PEPC activity. PA, phosphatidic acid; PC, phosphatidylcholine; PE, phosphatidylethanolamine; LPA, lyso-PA; PI, phosphatidylinositol4-P. Data represent mean ± SE of 3–6 different experiments. Statistically significant difference with respect controls are indicated by (^∗^) at *P* < 0.05, (^∗∗^) at *P* < 0.01, using the Dunnett test.

Due to the hydrophobic nature of phospholipids difficulties in their solubilization are acknowledged. Consequently, the apparent activity of every preparation was tested prior to its use. An aliquot of sp-PEPC was incubated in the presence or absence of the different lipids for analysis in 50 μl of a medium containing: 0.1 M Tris–HCl buffer (pH 8), 20% glycerol and 0.1 to 0.2 U of PEPC, at 30°C. Aliquots (5 μl) were taken to measure PEPC activity at pH 8.0 and 2.5 mM PEP, at the beginning and following 30 min of incubation. Activity was expressed as a percentage of the initial activity (see Figure 1C). Anionic but not neutral phospholipids may completely inactivate the enzyme within 30 min (Monreal et al., 2010).

### Proteolytic Assay in Standard Conditions

An aliquot of sp-PEPC containing co-purified protease(s) was incubated in 50 μl of a medium containing 0.1 M Tris–HCl buffer (pH 8) and 20% glycerol, at 30°C, in the presence or absence of the test lipids and/ or protease inhibitors. Aliquots were taken at different times during incubation, analyzed by SDS–PAGE (10% [w/v] acrylamide) and stained with Coomassie Blue or used for immunoblotting.

### Assessment of Protease Activity Using Fluorescent Substrates

Protease activity was assessed by measuring the hydrolysis of substrates containing the 7-amino-4-methyl coumarin (AMC) fluorophore in a microtiter plate format, at optimal pH according to the protease of interest. The standard assay volume was 100 μl containing 25 μl of sp-PEPC and the corresponding substrate added to a final concentration of 0.2 mM (Carrillo et al., 2011). Cathepsin B-like (CTB), L-like (CTL) and legumain (LEG) activities were assayed using *N*-Carbobenzoxyloxy-Arg-Arg-7-amido-4-methylcoumarin, (Z-RR-AMC); *N*-Carbobenzoxyloxy-Phe-Arg-7-amido-4-methylcoumarin (Z-FR-AMC) and *N*-Carbobenzoxyloxy-Ala-Ala-Asn-7-amido-4-methylcoumarin (Z-AAN-AMC) substrates, respectively, with a buffer containing 0.1 M citrate pH 6. Trypsin-like (TRY) and elastase-like (ELA) activities were assayed using Z-L-arginine-7-amido-4-methylcoumarin (ZLA-AMC) and MeOSuc-Ala-Ala-Pro-Val-7-amido-4-methylcoumarin (MeOS-AAPV-AMC) substrates, respectively, and a buffer containing 0.1 M glycine-NaOH pH 9.5. The leucine aminopeptidase (LAP) activity was assayed using L-Leu-7-amido-4-methyl coumarin (LL-AMC) substrate in a buffer containing 0.1 M Tris–HCl pH 7.5. All buffers contained 0.15 M NaCl and 5 mM MgCl_2_ (Carrillo et al., 2011). The reaction was incubated at 30°C for 24 h and fluorescence emitted was quantified with a 365 nm excitation wavelength filter and 465 nm emission wavelength filter in triplicate. Blanks were used to account for spontaneous breakdown of substrates and results were expressed as μmol min^-1^ ml^-1^. The system was calibrated with known amounts of AMC in a standard reaction mixture.

### Assay of PEPC Activity

PEPC activity was measured spectrophotometrically at pH 8.0 using the NAD-malate dehydrogenase-coupled assay at 2.5 mM PEP (Echevarria et al., 1994).

### *In vitro* Phosphorylation and PEPC Phosphorylation State

Aliquots of sp-PEPC were phosphorylated *in vitro* by the catalytic subunit of PKA from bovine heart according to the methods of Alvarez et al. (2003). The phosphorylation state of PEPC was determined using an L-malate test (Echevarria et al., 1994), where the malate inhibition of PEPC activity determined at suboptimal pH of 7.3 is expressed as an IC_50_ value. A high IC_50_ value is correlated to a high degree of PEPC phosphorylation (Echevarria et al., 1994).

### Electrophoresis and Immunoblotting

**Figure 2:**
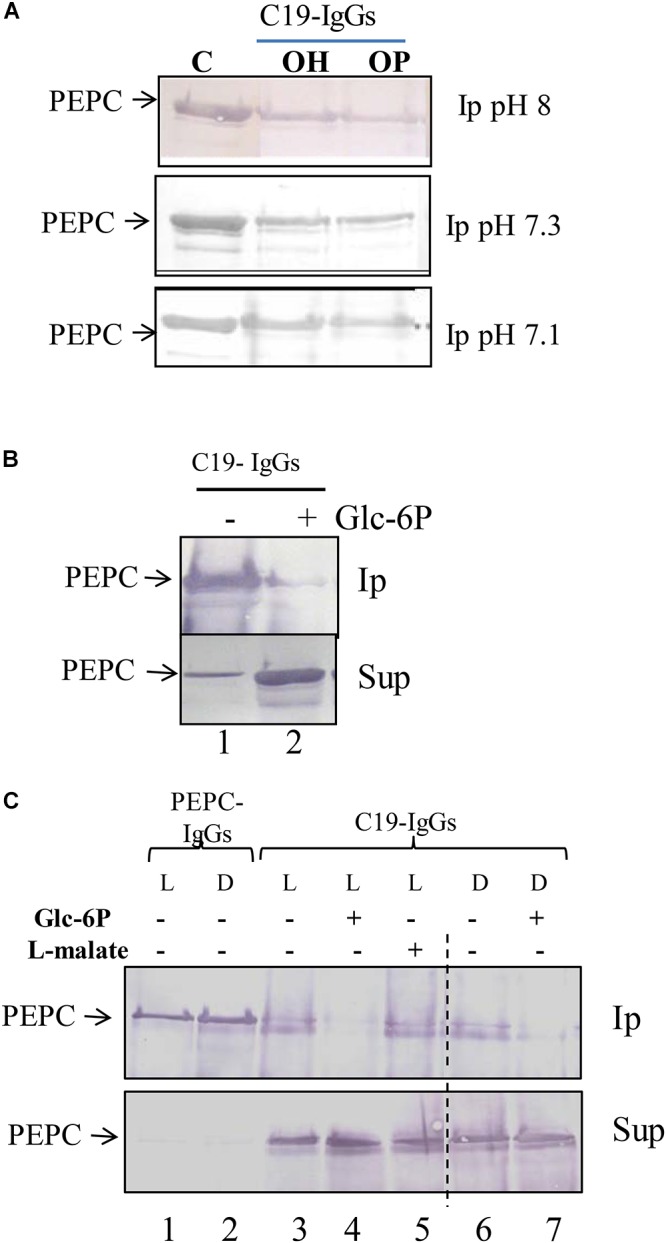
Effect of pH, enzyme phosphorylation state and Glc-6P on the exposure of the *C*-terminus of C_4_-PEPC. **(A)** Phosphorylated (OP) or non-phosphorylated (OH) sp-PEPC were incubated in 0.1 M Tris–HCl buffer at different pHs (8, 7,3 or 7,1). Following 30 min at 30°C, PEPC was immunoprecipitated with C19-IgG. Lane C, sp-PEPC prior to immunoprecipitation. Phosphorylated PEPC was obtained by *in vitro* phosphorylation with PKA as described in M&M. **(B)** Non-phosphorylated PEPC incubated with 5 mM Glc-6P for 30 min and then immunoprecipitated with C19-IgG. **(C)** Clarified protein extract from illuminated (L, phosphorylated-PEPC) or dark (D, non-phosphorylated-PEPC) sorghum leaves incubated in the presence of Glc-6P or L-malate and immunoprecipitated with PEPC-IgG (lanes 1, 2) or C19-IgG (lanes 3–7). Protein was detected on immunoblots using PEPC-IgG. Ip, immunoprecipitated; Sup, supernatant. The enzyme phosphorylation state was controlled via the L-malate sensitivity test (Table 1).

**Figure 3:**
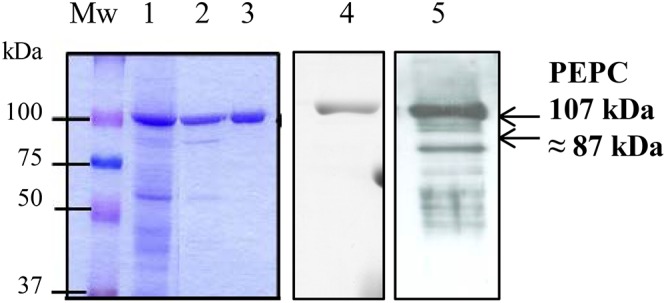
SDS–PAGE analysis of the sp-PEPC fraction during the purification process. PEPC (0.2 U) from the different purification steps were subjected to SDS–PAGE and staining with Commassie blue. Lane 1, clarified protein extract from dark-adapted sorghum leaves; Lanes 2, 3, chromatografhy on Hydroxyapatite and on anion-exchange, respectively. Lane 3 correspond to the final sp-PEPC preparation mainly used in the experiments. Lanes 1–3 contain 0.2 U of PEPC). Lane 4, immunoblot of lane 3 (0.05 U PEPC) detected using PEPC-IgG. Lane 5, immunoblot of sp-PEPC (0.1 U PEPC) following 3 months at –20°C.

**Figure 4:**
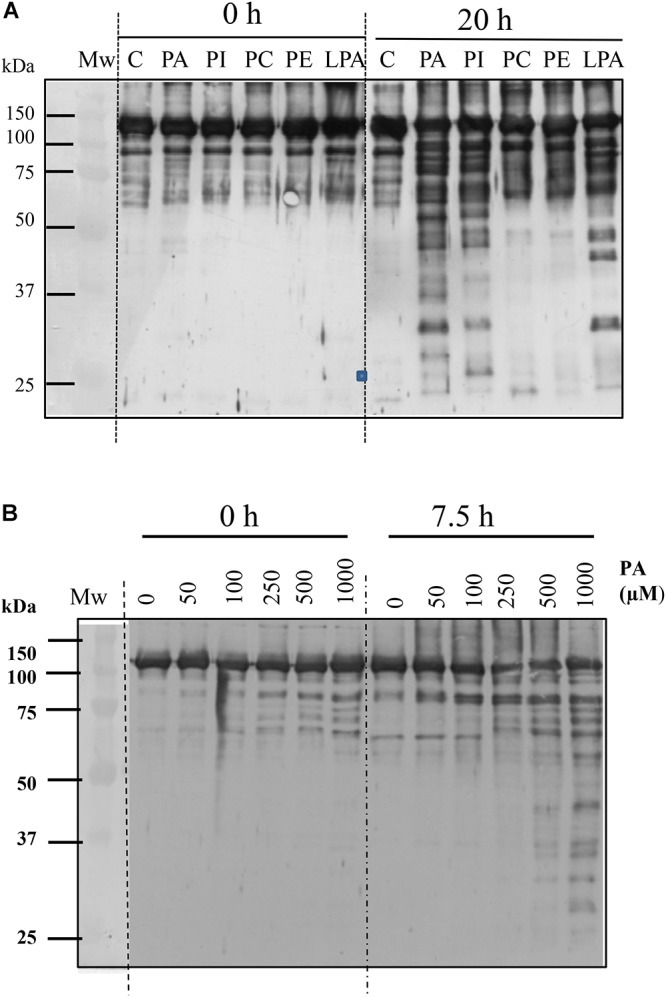
Anionic phospholipids promote proteolysis of C_4_-PEPC. **(A)** Sp-PEPC was incubated in the absence (lane C) or in the presence of 0.25 mM of the different phospholipids (lanes PA, PI, PC, PE, LPA) at 30°C as is described in M&M. **(B)** Range of PA concentration promoting PEPC proteolysis. A standard proteolytic assay was performed with increasing concentrations of PA18:1. At the indicated times, 0.05 U PEPC aliquots were removed and subjected to immunoblot. Bands were detected with PEPC-IgG.

**Figure 5:**
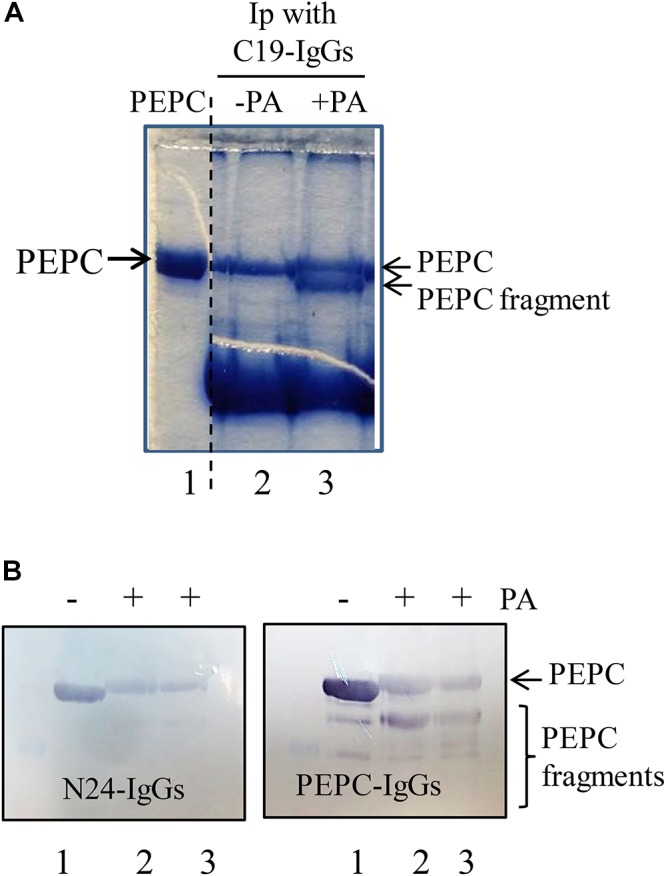
The *N*-terminal of PEPC is the initial target of PA-induced proteolysis. A standard proteolytic assay as is described in M&M was performed in presence or absence of 0.25 mM PA. **(A)** Coomassie blue stained PEPC samples exposed to PA for 60 min at 30°C and immunoprecipitated with C19-IgG. Lane 1, PEPC prior to immunoprecipitation. Lane 2, PEPC without PA. Lane 3, PEPC in presence of PA; **(B)** Immunoblot analysis of PEPC exposed to PA at different times and detected using N24-IgG or PEPC-IgG. Lane 1, PEPC control without PA. Lanes 2 and 3 in B, 5 or 7 h of incubation in presence of PA, respetively.

Protein samples were subjected to SDS–PAGE (10% [w/v] acrylamide) according to the method of Laemmli (Laemmli, 1970) at room temperature for 2 h at 100 V in a Mini-Protean ^®^III-2D cell (Bio-Rad). After electrophoresis, proteins on the gels were stained with Coomassie Blue R-250 or electroblotted onto a nitrocellulose membrane (N-8017, Sigma) at 10 V (5.5 mA cm^-2^) for 30 min in a semidry transfer blot apparatus (Bio-Rad Laboratories). Membranes were blocked in Tris-buffered saline (20 mM Tris–HCl and 0.15 mM NaCl [pH 7.5]) containing 5% (w/v) powdered milk, and bands were immunochemically labeled by overnight incubation of the membrane at 4°C in 20 ml of Tris-buffered saline containing specific antibodies. Subsequent detection was performed using a horseradish peroxidase conjugated antibody (Bio-Rad) by a peroxidase assay (Figure 1, 2, 3, 5) or by a chemiluminescence detection system (Super Signal West Dura Signal; ThermoFisher, Waltham, MA, United States) according to the manufacturer’s instructions (Figure 4).

### Immunoprecipitation

Immunoprecipitation of PEPC polypeptides following different experimental treatments was performed with relevant antibodies, as previously described by Osuna et al. (1999). Immunoprecipitates and supernatants were analyzed by SDS–PAGE and detected by Coomassie blue or immunoblotting.

### Statistical Analysis

All data in this report were obtained from at least three independent replicates. Data were analyzed by ANOVA and means were compared using the Duncan’s Multiple Range Test or the Dunnett test. A *P*-value of <0.05 was considered to be statistically significant. All statistical analyses were performed using SPSS statistic 34 software (IBM, Armonk, NY, United States).

## Results

### Phosphatidic Acid Inactivates PEPC Promoting Conformational Change

Sorghum C_4_-PEPC is a PA-binding protein that is inhibited in the presence of PA and other anionic phospholipids. The mechanism of this inhibition remains unknown, though it appears to be independent of allosteric regulators, substrate or pH (Monreal et al., 2010). To investigate the potential mechanism by which PA could inhibit C_4_-PEPC C19-IgG were utilized. Employing these antibodies allowed analysis of any potential changes in the conformation of the *C*-terminal portion of the protein, which is typically embedded within the protein subunit in the native protein conformation (Kai et al., 2003). To this end, 0.2 U of sp-PEPC were incubated with 0.25 mM PA for 30 min, at 30°C. After incubation 30 μg of C19-IgG were added and incubated 30 min more at room temperature. Finally, protein A (4% [w/v]) was added to the assay to precipitate the immune-complexes and analyzed by SDS–PAGE (Figure 1A) or immunoblotted and revealed with PEPC-IgG (Figure 1B). C19-IgG failed to immunoprecipitate the majority of native PEPC under standard conditions (Figure 1A,B, lane C2). This finding is in agreement with previous studies, demonstrating that the most abundant and active form of PEPC exhibits an embedded *C*-terminus conformation, which would not interact with the C19-IgG used (Alvarez et al., 2003; Kai et al., 2003). However, following incubation of sp-PEPC with PA, immunoprecipitation of PEPC was observed (Figure 1A,B, lanes PA). Both the anionic phospholipids PI and LPA have also been previously described as inhibitors of PEPC activity (Monreal et al., 2010), and treatment of sp-PEPC with either of these anionic phospholipids, in this study, similarly, resulted in exposure of the PEPC *C*-terminus to a similar extent as for PA (Figure 1A, PI and LPA; Figure 1B, LPA). In contrast to the activity of anionic phospholipids on the conformation of PEPC, both the non-anionic phospholipids, phosphatidylcholine (PC) and phosphatidylethanolamine (PE) minimally precipitated the enzyme, suggesting that they have little effect in inducing the conformational change of PEPC promoted by anionic phospholipids (Figure 1A,B, lanes PC and PE). Two controls were included in the experiment: an immunoprecipited PEPC with total PEPC-IgG (Figure 1A,B, lane C1), and PEPC heated at 60°C, 2 min, to completely expose the *C*-terminus previous to the incubation with C19-IgG (Figure 1A, lane 60°C; Alvarez et al., 2003). Finally, Figure 1C show the effectiveness of the phospholipids on PEPC activity in absence of C19-IgG. As expected (Monreal et al., 2010), complete inhibition of PEPC activity was observed in the presence of PA, PI or LPA, while PEPC activity was not affected by PC or PE (Figure 1C).

These results indicate that anionic phospholipids inactivate C_4_-PEPC promoting a conformational change, including exposure of the *C*-terminus. It is known that the carboxyl group of the *C*-terminal residue G960 is directed toward the PEP-binding site in the active conformation of PEPC and forms an ion pair with the side chain of residue R647, which is critical for maximal catalytic activity (Kai et al., 2003).

### Glc-6P Reverses the Enzyme Conformation to an Embedded *C*-Terminal State

Many elements have been shown to regulate PEPC activity, including pH, enzyme phosphorylation state, and the presence of specific metabolites. To determine whether any of these factors influence the exposure of the PEPC *C*-terminus, the enzyme conformation was assessed following incubation of sp-PEPC (0.1 U) under a number of different conditions for 1 h at 30°C (Figure 2). Post-treatment the PEPC enzyme was immunoprecipitated with C19-IgG, and analyzed by SDS–PAGE and immunoblot. Phosphorylated PEPC was obtained by *in vitro* phosphorylation with PKA and the enzyme phosphorylation state was controlled by use of the L-malate sensitivity test (see Table 1; Echevarria et al., 1994). Neither the phosphorylation state of PEPC nor changes in pH significantly modified the degree of exposure of the *C*-terminus (Figure 2A).

Interestingly, treatment of PEPC in which the *C*-terminal was exposed (Figure 2B, Ip, lane 1), with Glc-6P appeared to reverse the protein denaturation. In the presence of Glc-6P the C19-IgG failed to precipitate the enzyme (Figure 2B, Ip, lane 2) despite its extensive presence in the supernatant (Figure 2B, Sup, lane 2). Similar results were obtained by incubating desalted crude extracts from darkened or illuminated leaves with Glc-6P (Figure 2C, lanes 4 and 7). Conversely, in the presence of L-malate, a known negative effector of PEPC, (Chollet et al., 1996), the enzyme precipitated to a similar extent as the untreated control (Figure 2C, lane 5). Minimal difference in the level of PEPC immunoprecipitation was observed between the crude extracts obtained from illuminated (phosphorylated PEPC) or darkened (dephosphorylated PEPC) leaves (Figure 2C, lane 3 and 6). The phosphorylation state of PEPC from illuminated and darkened leaves was determined via the L-malate test (Echevarria et al., 1994) with leaves from illuminated plants showing twice the IC_50_ of those of darkened leaves (Table 1).

**Table 1:** The efficacy of *in vitro* phosphorylation of PEPC with PKA (sp-PEPC), and the *in vivo* phosphorylation state of PEPC in leaves exposed to light (phosphorylated PEPC) or dark (dephosphorylated PEPC) were determined via the L-malate sensitivity test as is described in M & M and Echevarria et al., 1994).

	Phosphorylation state of PEPC (IC_50_ for L-malate)
sp-PEPC	OH-PEPC	OP-PEPC
	0.64 ± 0.02	0.94 ± 0.07
Leaves Crude Extracts	Dark	Light
	0.31 ± 0.02	0.65 ± 0.03

*OH-PEPC, non-phosphorylated PEPC; OP-PEPC, phosphorylated PEPC. Values are mean ± SEM of triplicate assays*.

These results demonstrate that the conformational change promoted by the allosteric activator Glc-6P (Chollet et al., 1996) involves the *C*-terminus moving toward an embedded conformation, to stabilize the enzyme. The embedded and exposed *C*-terminal conformations of PEPC correlate well with high and low C_4_-PEPC enzyme activity. This integration of the *C*-terminus into the enzyme structure was seen to be induced in the presence of Glc-6P, and not by changes in the pH or phosphorylation state of the enzyme.

### Anionic Phospholipids (PA, PI, and LPA) Increase the Sensitivity of PEPC to Proteolysis

Regulation of PEPC proteolysis has not been extensively studied to date, and consequently little is known about the sensitivity of the different PEPC conformations to proteolysis. The observation that PA, PI, and LAP promoted a conformational change within the PEPC protein, including exposure of the *C*-terminus and inactivation of the enzyme, prompted us to explore whether this conformation was more sensitive to proteolysis.

C_4_-PEPC was obtained from dark-adapted sorghum leaves, by means of polyethylene glycol precipitation and chromatography on hydroxyapatite and anion exchange as described in M & M. This PEPC was pure, as shown by Coomassie-staining and SDS–PAGE (Figure 3, lanes 3). The preparation appeared stable at -20°C in a medium containing 0.1 M Tris–HCl, pH 7.5, 40% [v/v] glycerol for several weeks (Figure 3, lane 4). However, storage for 3 months at -20°C resulted in PEPC appearing to be partially proteolyzed (Figure 3, lane 5), providing evidence for the presence of proteases. We termed this fraction containing PEPC and proteases sp-PEPC within this study.

PEPC was shown to be very stable under standard incubation conditions, even for extended time periods of up to 20 h at 30°C (Figure 4, lanes C). However, incubation of sp-PEPC in the presence of 0.25 mM PA for 20 h resulted in proteolysis of the enzyme (Figure 4, lane PA). Comparable effects were observed for the physiological PA species, C18:1, and the water-soluble short-chain PA, C8:0 (Supplementary Figure S1). PA appeared effective in degrading PEPC at concentrations from approximately 0.25–1 mM PA (Figure 4B). However, it is difficult to accurately estimate the concentration of PA, as the physiological PA species (C18:1) is highly insoluble. The concentration of PA C8:0 (the water-soluble short-chain variant of PA) necessary to inhibit PEPC activity by 50% (IC_50_) was previously estimated by Monreal et al. (2010) and shown to be about 65 μM. Incubation of PEPC with the anionic phospholipids PI or LPA, also showed induction of the proteolysis of PEPC (Figure 4, lanes PI and LPA). Conversely, in the presence of the neutral phospholipids, PC or PE, PEPC maintained its integrity to a similar extent as the control (Figure 4, lanes PC and PE).

Collectively, these results provide evidence that PEPC can be targeted for proteolysis by interaction with anionic phospholipids, such as PA, and that the exposure of the enzyme’s *C*-terminus sensitizes the enzyme to this degradation. The contribution of the *C*-terminus of PEPC to the stability of the enzyme has previously referred by Xu et al. (2006). They found that mutagenic deletion of the last 19 residues of C_4_-PEPC from sorghum leaf (Leu-942 to G960), resulted in marked reductions in C_4_ enzyme accumulation when transformed onto PEPC *E. coli* cells (Xu et al., 2006).

Finally, PEPC activity was not restored following incubation with PA or LPA in the presence of proteases inhibitors (Supplementary Figure S2). This result suggests that the inhibition of PEPC activity in the early interaction with anionic phospholipids is due mainly to the conformational changes shown in Figure 1 and not to its proteolysis.

### The PEPC *N*-Terminal Is the Initial Target of PA-Induced Proteolysis

To determine whether the exposed *C*-terminus is an early target for proteolysis sp-PEPC samples were incubated in the presence and absence of 0.25 mM PA during 60 min at 30°C, followed by the addition of C19-IgG for 1.5 h at room temperature. Antibody-bound PEPC complexes were precipitated with protein A and analyzed by SDS–PAGE to reveal that, as expected, PA drives the exposure of the *C*-terminal thus allowing greater precipitation of the protein in the presence of PA. However, SDS–PAGE analysis of the pulled-down PEPC protein showed that a fraction of the protein appeared to be partially proteolyzed (Figure 5A, lane 3). As C19-IgG were used to bind the immuno-complexes, it is assumed that the *C*-terminal is present in this PEPC fragment and consequently it is not the target of the early proteolytic activity observed. This was confirmed using specific N24-IgG that recognize the *N*-terminus of the enzyme (the peptide [4ERHHSIDAQLRALAPGKVSEE24(YG)]). PEPC was incubated with PA and subsequently analyzed with the use of PEPC-IgG to show PEPC fragments of different size on immunoblots. However, analysis of these blots using the N24-IgG revealed that the various partially degraded PEPC fragments showed loss of the *N*-terminus at the beginning of the proteolytic process (Figure 5B, N24-IgG).

Taken together, these results suggest that the exposed *C*-terminus is not an early target for PA-induced proteolysis in sp-PEPC, rather, a fragment at the *N*-terminal portion of the protein is the initial site of protein degradation.

### PEPC Co-purifies With Papain-Like Proteases

**Figure 6:**
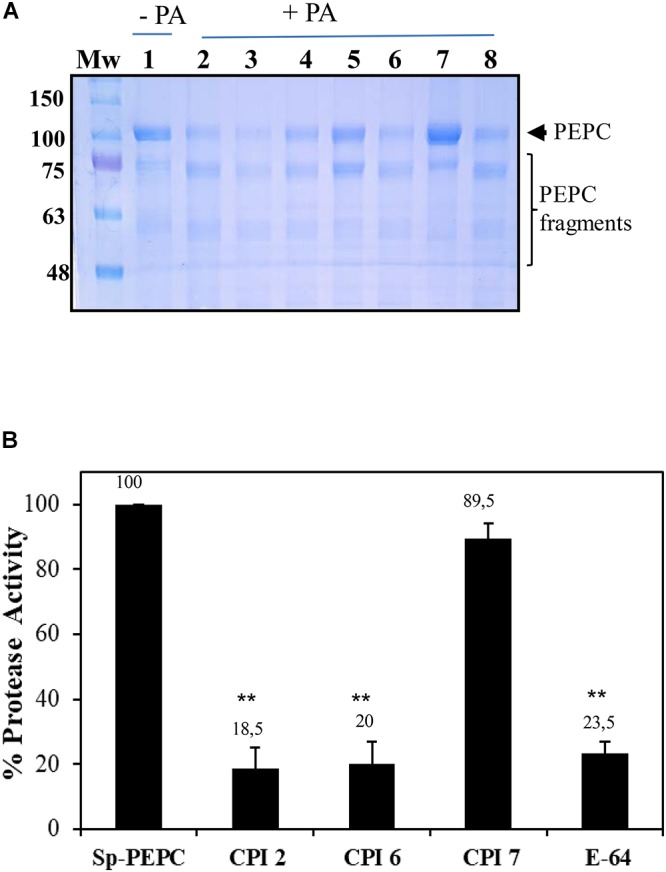
**(A)** sp-PEPC exposed to PA in the presence of protease inhibitors: sp-PEPC control (lane 1), no protease inhibitors (lane 2), 1 mM Bestatin (lane 3), 0.2 mM Leupeptin (lane 4), 0.065 mM Chymostatin (lane 5), 1 mM PMSF (lane 6), Sigma protease inhibitor cocktail containing AEBSF, 1,10-Phenanthroline, Pepstatin, Leupeptin, Bestatin and E-64 (lane 7) or 0.1 mM Aprotinin (lane 8). Following 20 h of incubation at 30°C, samples were subjected to SDS–PAGE and stained with Commassie Blue. **(B)** Cistatin and E-64 inhibit the protease activity present in sp-PEPC. Aliquots of sp-PEPC (25 μl) were incubated with 1 μM of recombinant purified cystatin (CIP) or E-64 in a 0.1 M citrate buffer (pH 6), 0.15 M NaCl and 5 mM MgCl_2_ for 10 min at 30°C. Protease activity was assayed by addition of 2 mM of the cathepsin L-specific substrate, Z-FR-AMC. The reaction was developed 5 h at 30°C. Protease activity is expressed as % with respect to the control in absence of inhibitor (sp-PEPC). Date are the mean ± SE of triplicate measurement and statistically significant difference with respect to the control are indicated by (^∗^) at *P* < 0.05, (^∗∗^) at *P* < 0.01, using the Dunnett test.

In order to identify the types of proteases that co-purified with PEPC as part of the sp-PEPC sample used in this study, a partial characterization of these enzymes was performed. Incubation of PEPC in the presence of PA and a variety of different protease inhibitors allowed identification of the protease activity. The protease activity in the sp-PEPC fraction appeared to be strongly inhibited by the inclusion of chymostatin (65 μM), a Cys/Ser protease type inhibitor (Figure 6A, lane 5); by E-64, a specific inhibitor of cysteine proteases (Figure 6B); and also, by the plant protease inhibitor cocktail (Figure 6A, lane 7) containing among others E-64. Other inhibitors such as bestatin (1 mM), leupeptine (0.2 mM), PMSF (1 mM) or aprotinin (100 μM), showed no significant inhibition on the protease activity present in sp-PEPC (Figure 6, lanes 3, 4, 6 and 8, respectively).

Commercial fluorescent substrates that are commonly used to identify proteases based on their specific activities, were utilized here to further characterize the proteases associated with sp-PEPC. Among the substrates assayed, Z-RR-AMC and Z-FR-AMC, substrates for cathepsin B-like (CTB) and cathepsin L-like (CTL) proteases, respectively, showed a large degree of degradation (Table 2). It is well known that the substrate Z-RR-AMC is highly specific for cathepsin B, while Z-FR-AMC is acted upon by cathepsin L, B, and F (Malagón et al., 2010). Proteolytic activity was detected throughout the range pH 5.5-8 in the presence of cathepsin B-like substrates (CTB), (Supplementary Figure S3). Cathepsin B-like and Cathepsin L-like proteases are cysteine proteases belonging to the papain-like family C1A (family C1, clan CA). This family is the most extensively studied and the most abundant among plant cysteine peptidases (Martínez and Díaz, 2008). Others substrates, such as LAP (Leucin aminopeptidase substrate), TRY (trypsin-like protease substrate), LEG (legumain protease substrate) and ELA (elastase-like protease substrate) were poorly proteolyzed or showed no proteolysis following incubation with sp-PEPC (Table 2).

**Table 2:** Identification of protease activities present in sp-PEPC fraction using specific protease fluorescent substrates.

Protease activity	pH^a^	Activity^c^ (μMol/min.ml sp- PEPC)
CTB	6.0	**43,3 ± 0,44 a**
CTL	6.0	**34 ± 3,98 b**
LEG	6.0	0,05 ± 0,02 c
TRY	9.5	2,73 ± 0,89 c
ELA	9.5	0,05 ± 0,02 c
LAP	7.5	5,65 ± 0,97 c

*CTB, cathepsin B-like; CTL, cathepsin L-like; LEG, legumain; TRY, trypsin; ELA, elastase; LAP, leucine aminopeptidase. ^*a*^pH measurements were the optimal for the different type of proteases activities ^*c*^. Values are mean ± SEM of triplicate measurements. Different letters denote statistically significant differences according to Duncan’s multiple range test, *p* < 0.05*.

Cystatin protease inhibitors are small proteins that have the ability to inhibit cysteine proteases from the papain-like family, C1A. These natural inhibitors play an important role in the regulation of endogenous cysteine protease activity (Martínez and Díaz, 2008; Díaz-Mendoza et al., 2014). Cystatin-2 (CPI-2) has been shown to significantly reduce cathepsin L-like and B-like activity in barley protein extracts, while CPI-6 is a specific inhibitor of cathepsin B. CPI-7 is reported to be inactive against a broad spectrum of proteases from barley (Martínez et al., 2009, 2012). Using the substrate with the broadest spectrum for cysteine proteases (Z-FR-AMC), the effect of the three recombinant cystatins (CPI-2, CPI-6, CPI-7) on protease activity in the sp-PEPC fraction was analyzed. Protease activity co-purified with PEPC appeared to be strongly inhibited by both CPI-2 and CPI-6, but not CPI-7 (Figure 6B).

Collectively, these results show that cysteine proteases are present in the sp-PEPC fraction and may be responsible for the proteolysis of PEPC in presence of PA. Among them, papain-like protease(s) (cathepsin B and L-like), from the C1A subfamily of the large Cys peptidase family are the major species present.

## Discussion

Little is known about the mechanism by which PEPC is proteolyzed. Several proteases have been described as possessing proteolytic activity that may target PEPC *in vitro*, though their *in vivo* activity often remains unexplored. Within this study, we describe a relationship between the interaction of C_4_-PEPC with anionic phospholipids and the proteolysis of this enzyme. The results presented here demonstrate that PA changes the conformation of the enzyme, resulting in the exposure of the *C*-terminus to allow its precipitation by C19-IgG (Figure 1). This conformation was found to be sensitive to proteolysis. Incubation of PEPC in presence of PA increased the proteolysis of the enzyme (Figure 4). The conformational changes and the induction of proteolysis were also promoted by other anionic phospholipids, such as PI or LPA, leading the exposure of the *C*-terminus (Figure 1) and the inactivation of the enzyme (Monreal et al., 2010, and Figure 1C). Neutral phospholipids, such as PC or PE, minimally influenced conformational change in PEPC or any resultant proteolysis. These results represent the first *in vitro* evidence of anionic phospholipids being identified as possible molecules that can trigger the proteolysis of C_4_-PEPC. As we have previously described, a portion of intracellular PEPC *in vivo* interacts with the cellular membranes, and this membrane-associated PEPC appears partially proteolyzed (Monreal et al., 2010). Hence, the results presented in this work support the hypothesis that anionic phospholipids could recruit PEPC to the cell membranes for its proteolysis. A previous study by Wu and Wedding (1992), lends weight to this idea, as it discusses the *in vitro* inactivation of maize leaf PEPC by its binding to the outer chloroplast membranes. Association of PEPC with the outer chloroplast membrane has also been observed by additional immunolocalization studies (Perrot-Rechenmann et al., 1982). Furthermore, bacterial- and plant-type PEPC isozymes from developing castor oil seeds have been seen to interact *in vivo* and associate with the outer surface of mitochondria (Park et al., 2012).

Other metabolic enzymes, such as Rubisco, have been reported to travel to the vacuole to be proteolyzed. The proteolysis of Rubisco has been extensively studied and different mechanisms have been described, whereby Rubisco is packed into senescence-associated lytic vacuoles (SAV) that are rich in cysteine proteases, or into Rubisco-containing bodies (RCBs) that drive the enzyme to the vacuole to be digested. Also, Three different autophagic vesicles, in addition to the process of chlorophagy, have also been reported to degrade Rubisco (Ono et al., 2013; for a further revision Otegui, 2018). Rubisco, represents an important source of nitrogen in senescence conditions and is the main target for proteases (Ono et al., 2013). However, in C_4_ plants, photosynthetic C_4_-PEPC is highly abundant and competes with the Rubisco for nitrogen. PEPC constitutes about a 15% of total soluble protein in C_4_ plants (maize). The ratio of phosphoenolpyruvate carboxylase to Rubisco in C_4_-plant is about 2:1, whereas it is of 0.1:1 in C_3_-plants (O’Leary, 1982). Consequently, in a similar manner as Rubisco, C_4_-PEPC may serve as another important target for proteases in senescent leaves. In fact, PEPC is degraded in sorghum plants treated with LiCl and in old leaves of control plants (García-Mauriño et al., 2003; Monreal et al., 2007). Taken together, these results suggest a potential mechanism by which anionic phospholipids are able to inactivate PEPC and target the enzyme for proteolysis. This could be of interest in a physiological context where the degradation of C_4_-PEPC is necessary (such as in senescence, stress or photo-damaging radiation) in parallel with other abundant metabolic enzymes such as Rubisco in order to recycle key nutrients. In agreement with this concept, PA transiently accumulates in plant cells within minutes of applying a wide array of stress conditions (O’Leary et al., 2011). Whether the association of C_4_-PEPC with cell membranes acts as the initiation of a proteolytic pathway, similar to those operating for Rubisco, or it interact with the membrane of the chloroplast to be engulfed in a chlorophagic process (Otegui, 2018) remains to be investigated. Further experiments will be necessary to study the possible association and/or location of GFP-fused C_4_-PEPC in vesicles that are eventually transported into the vacuole for degradation, as occurs with Rubisco (Otegui, 2018). Finally, investigation are need to know how PEPC activity or cellular location is influenced by changes in anionic membranes phospholipids.

Sorghum C_4_-PEPC is a PA-binding protein that is inhibited in the presence of anionic phospholipids, PA, PI and LPA. In this work we outlined the mechanism by which PA may inactivate PEPC. This mechanism is based upon the conformational change observed in PEPC that is induced by PA and other anionic phospholipids, in which the C-terminal is exposed. The carboxyl group of the *C*-terminal residue G960 forms part of the active site of PEPC (Kai et al., 2003). Movement of this amino acid impacts on the active conformation of PEPC, driving it to inactivation. Previous reports of the relevance of the *C*-terminus in determining PEPC activity and stability support these findings (Xu et al., 2006).

Glc-6P is a known allosteric activator of PEPC (Chollet et al., 1996). Higher plant PEPCs are generally inhibited by L-malate and activated by Glu-6P. During C_4_ photosynthesis PEPC has to fix bicarbonate in the presence of its allosteric inhibitor L-malate. The concentration of L-malate in the mesophyll cell is high enough to block PEPC activity. In this context, PEPC activity is activated *in vivo* by the interplay of Glc-6P and the phosphorylation state of the enzyme (Echevarria et al., 1994). Our findings demonstrate that Glu-6P is able to change the conformation of PEPC to promote the native embedded *C*-terminus conformation (Figure 2B) that activates and stabilizes the enzyme against proteolysis. This finding supports the fact that the embedded and exposed conformations of the *C*-terminus correlate well with the active and inactive state of the enzyme, respectively. The influence of the conformational state of PEPC on its proteolysis has been previously demonstrated in maize PEPC with the use of *in vitro* trypsin treatment. For maize PEPC, the activation state of the enzyme during the trypsin attack is dependent upon the presence of a catalytic effector or substrates (PEP+Mg, Glc-6P or L-malate) during the treatment (Maralihalli and Bhagwat, 2001).

Proteolysis of the *N*-terminal of PEPC during purification has been extensively reported. PEPC from maize leaves (C_4_-PEPC photosynthetic form) was purified as a double band of 109 and 105 kDa, where the105 kDa species represented a proteolyzed version of the 109 kDa protein in which L-malate sensitivity was lost (McNaughton et al., 1989). The presence of the protease inhibitor chymostatin throughout the enzyme preparation was found to be essential for the maintenance of the L-malate sensitivity of PEPC during purification. This result suggests that chymostatin acts to protect the enzyme against cleavage of a peptide bond close to the N-terminal domain, which includes the phosphorylation site and sensitivity to malate (McNaughton et al., 1989). Other protease inhibitors, such as Benzamidine and PMSF, failed to prevent this proteolysis. Indeed, similar results were reported by Baur et al. (1992), who demonstrated that when purified in the absence of the protease inhibitor chymostatin, PEPC from *Mesembryanthemum crystallinum* lost an *N*-terminal sequence of 128 amino acids. This proteolyzed enzyme could not be phosphorylated *in vitro*, which is in agreement with the *N*-terminal domain containing the phosphorylation motif (Baur et al., 1992). In both cases the proteolysis of this *N*-terminal fragment affected L-malate sensitivity, but did not impact upon the final specific PEPC activity. Furthermore, it has been reported that the proteolytic cleavage of the 22 amino acids at the *N*-terminal of C_4_-PEPC markedly decrease its malate sensitivity (Duff and Chollet, 1995; Chollet et al., 1996). The *N*-terminal proteolysis of the non-photosynthetic PEPC (C_3_-PEPC) has also recently been investigated, demonstrating that proteolysis of this C_3_-PEPC is performed by an endogenous asparaginyl endopeptidase, which hydrolyzes a polypeptide of approximately 120 amino acids from the *N*-terminus of the castor oil seed PEPC (Crowley et al., 2005). Pioneering studies have shown that the *in vivo* processing of PEPC during the germination of castor oil seeds includes monoubiquitination, with both monoubiquitinated and deubiquitinated subunits being *N*-terminally truncated by 19 amino acids. Moreover, processing of the non-ubiquitinated subunits was induced by truncation of 4 amino acids at the *N*-terminus (Uhrig et al., 2008; O’Leary et al., 2011).

In this study, we have identified and partially characterized a protease activity that co-purified with PEPC. This enzyme showed sensitivity to the Ser/Cys-protease inhibitors, chymostatin and E-64. A more extensive characterization using specific commercial fluorescent substrates, commonly used to identify proteases based on their specific activity, was carried out. Among the substrates assayed, CTB and CTL (substrates for cathepsin B-like and cathepsin L-like proteases, respectively) were efficiently degraded by the proteolytic activity present in the sp-PEPC. Moreover, the protease activity was inhibited by CPI-2 and CPI-6, cystatin inhibitors specific to cathepsin-like proteases L and B, but was not affected by CPI-7 (Figure 6B), which has been shown to be inactive against a broad spectrum of proteases in barley plants (Martínez et al., 2009). In agreement with previous studies on the degradation of PEPC, we have shown that proteolysis performed by the identified cysteine protease activity resulted in the initial loss of a fragment from the *N*-terminal of C_4_-PEPC (Figure 5).

Our results show that cysteine proteases are the main source of protease activity present in the sp-PEPC fraction, with papain-like proteases (cathepsin B and L) representing the majority of co-purified proteases. To gain further information concerning the role of this protease activity, including its physiological relevance to PEPC turnover within the context of C_4_-photosynthesis in normal and stressed conditions, it is essential that the protein is purified and the gene identified. Aditionally, bimolecular fluorescence complementation (BiFC) experiments will be necessary to verify the association of the proposed proteases with PEPC *in vivo*.

## Conclusion

Here we have outlined the mechanism by which PA and other anionic phospholipids inhibit C_4_-PEPC activity in sorghum. Large conformational changes in the C_4_-PEPC protein complex, evidenced by the exposure of the *C*-terminus, that remains embedded within the protein in the active conformation, are responsible for the inhibition of C_4_-PEPC. It has also been demonstrated that the conformational change promoted by anionic phospholipids is very sensitive to proteolysis by proteases that co-purify with C_4_-PEPC. These proteases have been partially characterized and found to largely consist of cysteine proteases, with an abundance of cathepsin B and L. Finally, this work represents the first demonstration that anionic phospholipids could trigger one of the different proteolytic pathways operating to degrade C_4_-PEPC.

## Author Contributions

JG, J-AM, CE, SG-M conceived the idea and led the study design. JG, J-AM, RÁ carried out the experiments. ID provided fluorescent substrates of proteases and cystatin for the experiments of proteases characterization which were performed in her laboratory by JG. CE wrote the manuscript.

## Conflict of Interest Statement

The authors declare that the research was conducted in the absence of any commercial or financial relationships that could be construed as a potential conflict of interest.
