# Supplementary material for: Anionic Phospholipids Induce Conformational Changes in Phosphoenolpyruvate Carboxylase to Increase Sensitivity to Cathepsin Proteases
Source: Front Plant Sci. 2019 May 9;10:582. doi: 10.3389/fpls.2019.00582 (PMC6521631; doi:10.3389/fpls.2019.00582)
Supplement: Supplementary file 1 [file Image_1.PDF]

Supplementary Material

Anionic phospholipids induce conformational changes in phosphoenolpyruvate carboxylase to increase sensitivity to cathepsin proteases.

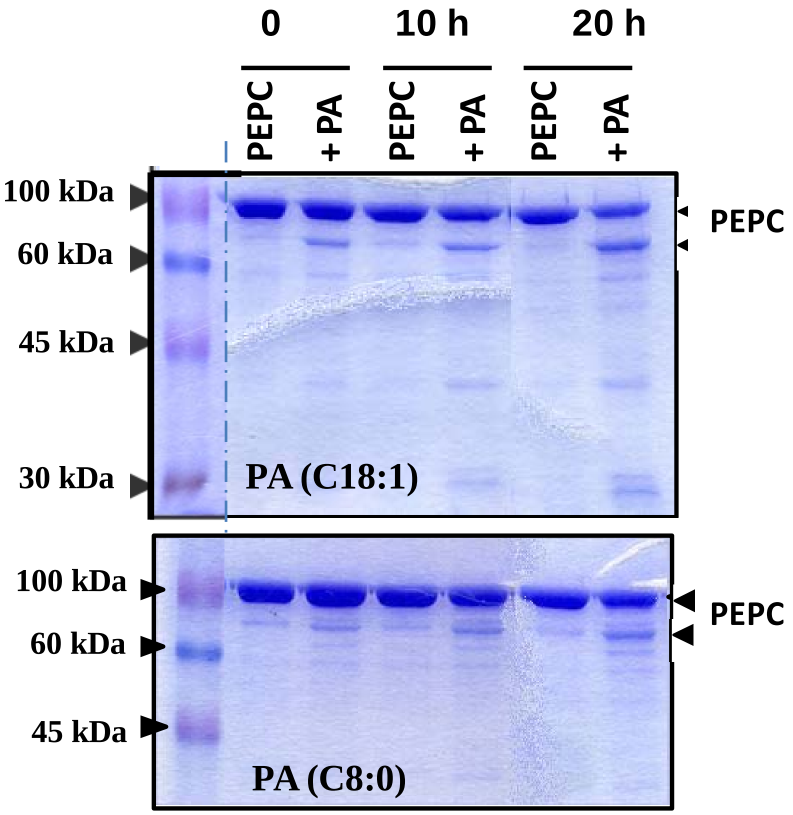

Supplementary Figure S1. PA appeared effective in degrading PEPC and comparable effects were observed for the physiological PA species, C18:1, and the water-soluble short-chain PA, C8:0. Sp-PEPC was incubated in the presence of 0.25 mM at 30 ºC as is described in M&M. At the indicated times, 0.05 U PEPC aliquots were removed, subjected to SDS-PAGE and stained with Commassie Blue.

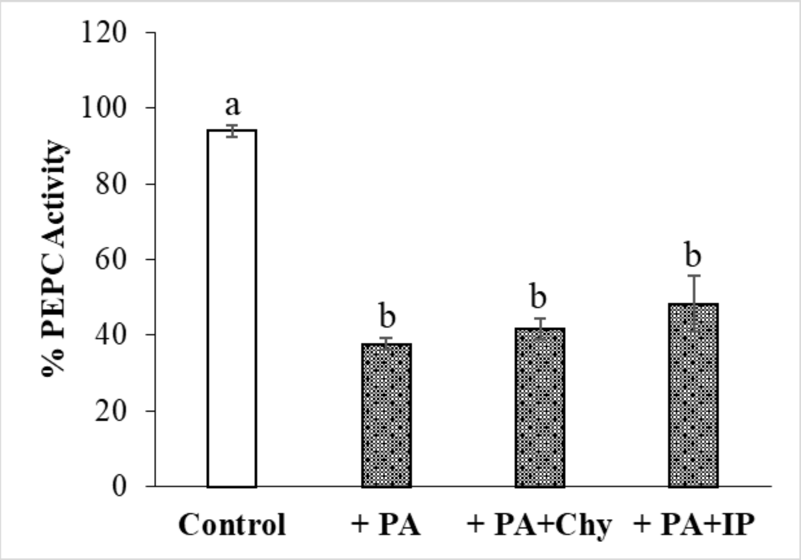

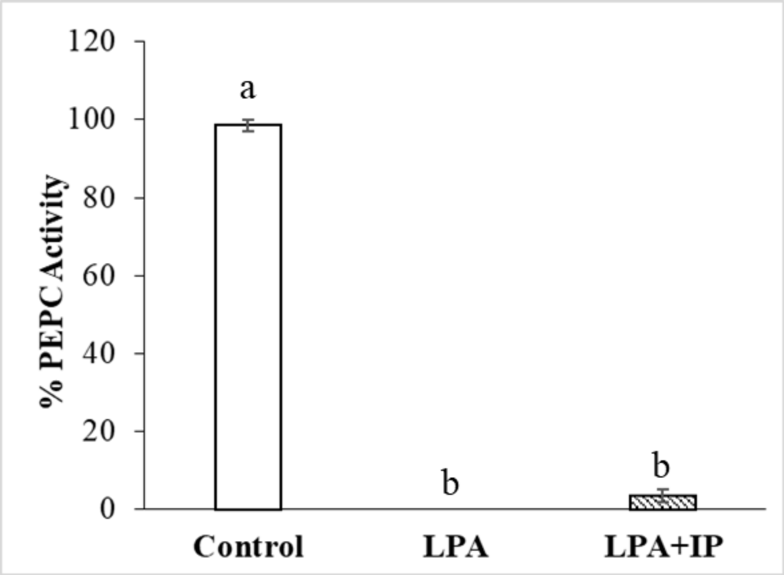

Supplementary Figure S2: PEPC activity was not restored following incubation for 30 min with PA or LPA in the presence of proteases inhibitors. Chy, Chymostatin; IP, Sigma protease inhibitor cocktail containing AEBSF, 1,10-Phenanthroline, Pepstatin, Leupeptin , Bestatin and E-64. Data shown are the mean ± SE (n=3). Different letters indicate significant differences according to Tukey B multiple range test, $p<0.05$.

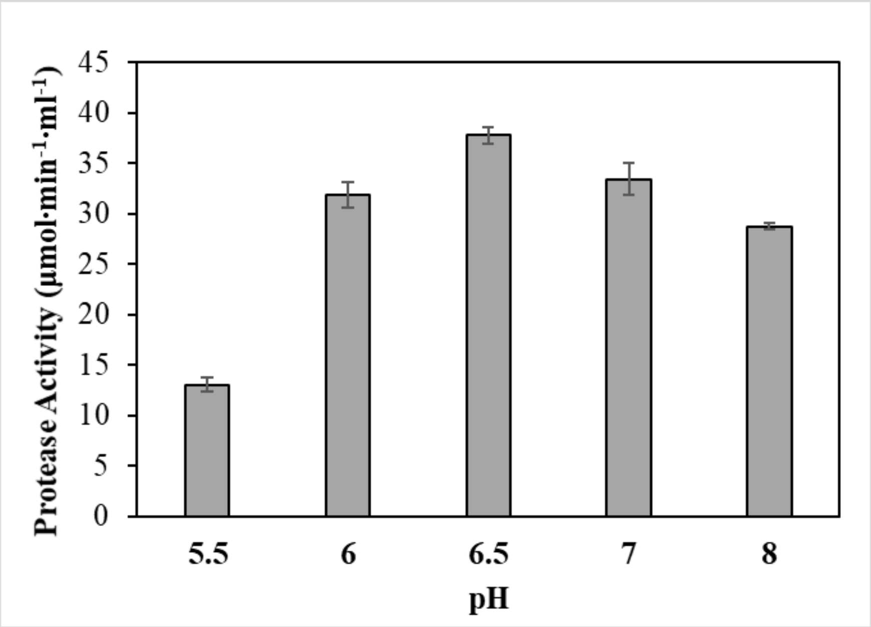

Supplementary Figure S3. Protease activity present in sp-PEPC is active over a wide pH range. Sp-PEPC was incubated with the cathepsin B-specific substrate, Z-RR-AMC, to assess the sensitivity of protease activity over the examined pH range. Series of buffers used: 0.1 M citrate (pH 5-6), 0.1 M phosphate (pH 6.5) and 0.1M Tris-HCl (pH 7-8). All buffers contained 0.15 M NaCl and 5 mM of $MgCl_2$. Date are the mean±SE of triplicate measurement.
